# Potential Protective Mechanisms of S-equol, a Metabolite of Soy Isoflavone by the Gut Microbiome, on Cognitive Decline and Dementia

**DOI:** 10.3390/ijms231911921

**Published:** 2022-10-07

**Authors:** Akira Sekikawa, Whitney Wharton, Brittany Butts, Cole V. Veliky, Joshua Garfein, Jiatong Li, Shatabdi Goon, Annamaria Fort, Mengyi Li, Timothy M. Hughes

**Affiliations:** 1 Department of Epidemiology, School of Public Health, University of Pittsburgh, Pittsburgh, PA 15213, USA; 2 School of Nursing and Medicine, Emory University, Atlanta, GA 30322, USA; 3 Department of Medicine, University of Pittsburgh School of Medicine, Pittsburgh, PA 15213, USA; 4 Department of Internal Medicine, Wake Forest School of Medicine, Winston-Salem, NC 27157, USA

**Keywords:** S-equol, arterial stiffness, white matter lesions, Alzheimer’s disease and related dementias, vascular contributions to cognitive impairment and dementia, estrogen receptor-β agonist

## Abstract

S-equol, a metabolite of soy isoflavone daidzein transformed by the gut microbiome, is the most biologically potent among all soy isoflavones and their metabolites. Soy isoflavones are phytoestrogens and exert their actions through estrogen receptor-β. Epidemiological studies in East Asia, where soy isoflavones are regularly consumed, show that dietary isoflavone intake is inversely associated with cognitive decline and dementia; however, randomized controlled trials of soy isoflavones in Western countries did not generally show their cognitive benefit. The discrepant results may be attributed to S-equol production capability; after consuming soy isoflavones, 40–70% of East Asians produce S-equol, whereas 20–30% of Westerners do. Recent observational and clinical studies in Japan show that S-equol but not soy isoflavones is inversely associated with multiple vascular pathologies, contributing to cognitive impairment and dementia, including arterial stiffness and white matter lesion volume. S-equol has better permeability to the blood–brain barrier than soy isoflavones, although their affinity to estrogen receptor-β is similar. S-equol is also the most potent antioxidant among all known soy isoflavones. Although S-equol is available as a dietary supplement, no long-term trials in humans have examined the effect of S-equol supplementation on arterial stiffness, cerebrovascular disease, cognitive decline, or dementia.

## 1. Introduction

Nutrition is considered an important modifiable factor in cognitive dysfunction and Alzheimer’s disease and related dementias (ADRD). High adherence to a Mediterranean diet is associated with a reduced risk of global cognitive decline [1]. The MIND (Mediterranean DASH diet Intervention for Neurodegenerative Delay) diet is associated with slower cognitive decline [2]. Evidence suggests that certain nutrients, such as polyphenols and long-chain n-3 fatty acids, can benefit cognitive function due to their antioxidant, anti-inflammatory, and other properties [3,4,5,6,7]. One such class of polyphenolic compounds is soy isoflavones, of which two major constituents are daidzein and genistein. Soy isoflavones are structurally similar to estradiol [8]. While estradiol exerts its biological actions through binding to both estrogen receptor α (ERα) and estrogen receptor β (ERβ), soy isoflavones are ERβ-selective agonists [9]. Soy isoflavones possess antioxidant [10], anti-inflammatory [11], anti-atherosclerotic [12], and vasodilatory [13] properties, with associated health benefits, including those related to cognition [14].

Soy isoflavones are regularly consumed in Japan and other East Asian countries, where their dietary intake is 25–50 mg/day [14,15]. In contrast, consumption is minimal in the US and other Western countries, i.e., <2 mg/day [14,16]. Recent studies in Japan reported that a diet high in soy isoflavones is inversely associated with incident cognitive impairment [17] and dementia [18]. However, a randomized controlled trial (RCT) of soy isoflavones in the US, the Women’s Isoflavone Soy Health (WISH) Trial, showed no significant effect of soy isoflavones on global cognition [19]. This discrepancy between the results in Japan and the US may be due to the difference in S-equol-producing capability. S-equol, a metabolite of a soy isoflavone daidzein, transformed by the gut microbiome, is the most bioactive among all soy isoflavones and their metabolites [20]. After consuming soy isoflavones, 50–70% of Japanese convert daidzein to S-equol. Because dietary intake of soy isoflavones in the US is minuscule [14,16], the rate of S-equol producers is estimated from RCTs of soy isoflavones; 20–30% of individuals assigned to supplementation of soy isoflavones (ranging from 44 to 210 mg/day) produce S-equol [21,22,23,24,25,26]. The difference in the rate of equol producers between Asian and Western countries is believed to be due to the difference in the gut microbiome [27,28,29,30] but not genetics [31,32,33]. A subgroup analysis in WISH showed that S-equol producers had improved cognition compared to the control group [19]. A cross-sectional study in older adults in Japan showed that S-equol production was significantly inversely associated with mild cognitive impairment (MCI) [34]. These results suggest that S-equol may be the key factor for the reported cognitive benefit of soy isoflavones. Here, we provide a narrative review of the bioavailability of S-equol compared to daidzein and genistein, epidemiological and clinical evidence to support that S-equol has cognitive benefits, potential mechanisms of the beneficial effects of S-equol on cognition and neurodegeneration, ERβ and cognition, and neuroprotection by ERβ agonists.

## 2. Soy Isoflavones and Conversion of Daidzein into S-equol

**Figure 1:**
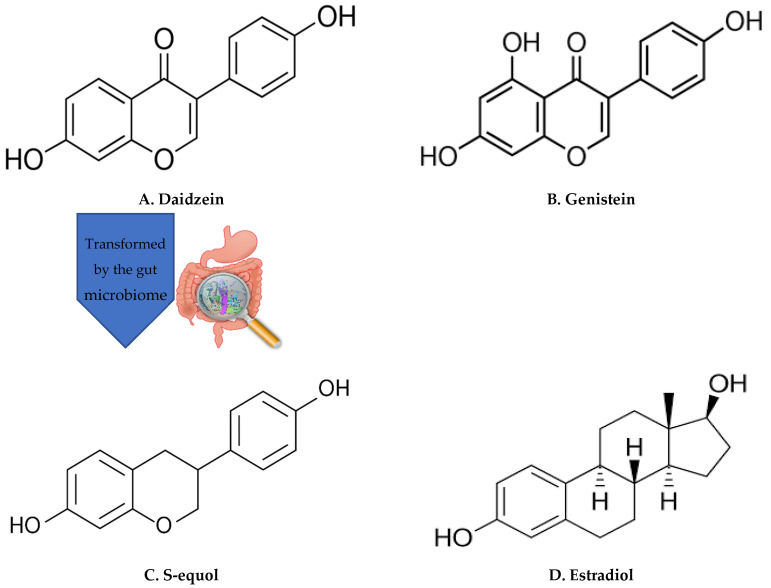
Chemical structures of daidzein, genistein (two major soy isoflavones), equol, and estradiol. Daidzein and genistein are two major soy isoflavones and comprise >95% of their dietary source. Soy isoflavones and S-equol are structurally similar to estradiol. S-equol is a metabolite of daidzein, transformed by the gut microbiome. Daidzein is reduced to S-equol through the intermediate dihydrodaidzein and then converted by deoxygenation to yield S-equol. [27] Daidzein, genistein and equol are structurally similar to estradiol (images of chemical structures are from Wikimedia Commons and Pixabay).

Soy isoflavones are polyphenols and phytoestrogens, plant-derived compounds structurally similar to 17β-estradiol (Figure 1). Therefore, many biological actions of soy isoflavones are exerted through their binding to ERs. There are two subtypes of ERs: ERα, which is present mainly in the mammary gland, ovary, liver, and bone, and ERβ, which is found in the colon, adipose tissue, and immune system. ERα and ERβ are expressed in the central nervous and cardiovascular systems [8]. Activation of ERα is associated with proliferative responses in the mammary gland, e.g., breast cancer and uterus. While estradiol binds to ERα and ERβ, soy isoflavones preferentially bind to ERβ [9]. Thus, soy isoflavones could potentially have beneficial estrogen-like effects with reduced side effects [8].

Soy isoflavones in soybeans are present as glycosides, bound to a sugar molecule. The sugar molecule is hydrolyzed upon digestion or fermentation, leaving an isoflavone aglycone, e.g., daidzein and genistein. The isoflavone aglycones are more readily absorbed than glucosides in humans [14]. The conversion of daidzein into S-equol (Figure 1) takes place in the intestine by the action of the gut microbiome [35]. Several bacteria that transform daidzein into S-equol have been isolated from the feces of animals and humans. These bacteria are anaerobes, rod-shaped, and Gram positive. In vitro experiments have succeeded in producing S-equol from daidzein-rich soy germ using bacteria [36]. Thus, S-equol has become available as a nutraceutical [37].

## 3. Bioavailability of S-equol as Compared to Daidzein and Genistein

### 3.1. Bioavailability in the Circulation

S-equol has the highest bioavailability among all soy isoflavones and their metabolites [20,38]. Preclinical studies show that the affinity of S-equol to ERβ is 18-fold higher than its precursor, daidzein, and S-equol’s affinity is similar to genistein [39]. Unlike daidzein or genistein, S-equol undergoes little biotransformation in the circulation [40,41]. Moreover, the biological activity of S-equol is enhanced by its reduced binding to serum proteins [41]. Almost 50% of S-equol circulates in the free form compared to less than 20% for daidzein and genistein [41,42].

### 3.2. Permeability to the Gastrointestinal Tract and Blood–Brain Barrier

As described later, in vivo studies report that S-equol and soy isoflavones have beneficial effects on the central nervous and cardiovascular systems. The critical question is whether these compounds can cross the gastrointestinal (GI) tract and the blood–brain barrier (BBB). To answer this question, recently, Johnson et al. used the SwissADME and the high-throughput parallel artificial membrane permeability assay (PAMPA) to determine the permeability properties of S-equol, genistein, daidzein, and other polyphenols through the GI tract and BBB. The SwissADME is a computational predictive software that computes key physicochemical, pharmacokinetic, drug-like, and related parameters for one or multiple molecules [43]. PAMPA is a robust, versatile method for predicting the passive permeability of compounds through the GI tract and BBB [44]. PAMPA uses a combination of phospholipids specific to the tested membrane and a microfiber filter to simulate the biological membrane. SwissADME showed that S-equol, genistein, and daidzein have high gut absorbance and that S-equol and daidzein but not genistein have BBB permeability. PAMPA showed that S-equol has higher GI tract and BBB permeability than daidzein or genistein. Thus, S-equol exhibits high permeability in both the GI tract and BBB.

## 4. Evidence from Epidemiological and Clinical Studies Evaluating the Cognitive Benefits of S-equol

Recently, two well-designed prospective cohort studies in Japan reported that soy isoflavones and a diet rich in soy conferred cognitive benefits. The National Institute for Longevity Sciences—Longitudinal Study of Aging (NILS-LSA) followed a population-based cohort of 776 men and women (mean age of 68 at baseline) for 8 years [17]. Those with Mini-Mental State Examination (MMSE) scores ≤23 were excluded at baseline. During the mean follow-up of 8 years, MMSE was administered 5 times on average. Incident cognitive impairment was defined as an MMSE score ≤23. Multivariable-adjusted hazard ratio (HR) of incident cognitive impairment associated with one standard deviation (SD) increase in dietary intake of soy isoflavones was 0.55 (95% confidence interval (CI): 0.23, 0.93) in women and 0.89 (95% CI: 0.58, 1.37) in men. The Hisayama Study followed a population-based cohort of 1006 dementia-free men and women (mean age of 68 at baseline) for 16 years. During the follow-up, 271 subjects developed dementia. A diet rich in soy, soy isoflavones, and vegetables was significantly inversely associated with incident dementia (multivariable-adjusted HR in the highest quartile of intake was 0.66 (95% CI: 0.46, 0.95) compared to the lowest quartile (*p* for trend = 0.02) [18]. However, S-equol was not measured in either study.

A recent cross-sectional study in Japan of 152 subjects (mean age of 69) reported that S-equol producers had significantly higher cognitive scores and lower prevalence of MCI than non-producers and that equol-producing status was a significant determinant of MCI in the multivariable analysis [34]. Other studies in Asians (Singapore [45], Chinese [46], and Asian Americans [46]) reported no significant association of dietary intake of soy isoflavones with cognitive impairment. However, their dietary intake of soy isoflavones (3–9 mg/day) was lower in these studies than in Japan (25–50 mg/day) and their S-equol-producing status was not reported.

Since soy isoflavones are phytoestrogens, most RCTs of soy isoflavones on cognition have been conducted in women, especially post-menopausal women. Our recent systematic review and meta-analysis of 16 RCTs of soy isoflavones on cognition (total number of participants = 1384 (134 male and 1252 female), mean age of 60, dose of soy isoflavones ranging from 60 to 160 mg/day, median duration of intervention of 17 weeks) showed that supplementation of soy isoflavones significantly improved overall cognitive function (standard mean difference 0.19 (95% CI: 0.07–0.32)) and memory (standard mean difference 0.15 (95% CI: 0.03–0.26) in adults [47]. Subgroup analysis showed no statistically significant difference in the duration of the intervention (<6 vs. ≥6 months), dose (<100 vs. ≥100 mg/day), or age (<60 vs. ≥ 60 years). However, the number of participants in most of these RCTs was small (<100), with the duration of intervention less than 1 year. The WISH trial, which had the largest number of participants with the most extended intervention period in these 16 RCTs, did not show significant improvements in overall cognition [19].

The WISH trial, an RCT evaluating the impact of 91 mg/day of soy isoflavones for 2.5 years on cognition among 350 US women (mean age of 61), showed that soy isoflavones did not improve global cognition but improved visual memory [19]. The subgroup analysis by S-equol-producing status did show that only 26% were S-equol producers among women assigned to soy isoflavones and that global cognition marginally significantly improved in S-equol producers compared to the placebo group (mean standardized changes in global cognition (standard error), 0.65 (0.18) vs. 0.31 (0.08) for equol producers and placebo group, respectively, *p* = 0.08). These results suggest that S-equol rather than soy isoflavones slows cognitive decline.

## 5. Potential Protective Mechanisms of S-equol for Cognitive Decline and ADRD

While amyloid-β deposition and phosphorylated tau are major therapeutic targets being tested in recent RCTs [48] or developed [49] for Alzheimer’s disease (AD), vascular, inflammatory, immune, metabolic, and other factors are also involved in pathophysiology of AD/ADRD. Among these, vascular contributions to cognitive impairment and dementia (VCID) [50] are an emerging and promising approach to dementia prevention. This is further evidenced by the Systolic Blood Pressure Intervention Trial (SPRINT) MIND RCT, which reported that intensive blood pressure treatment lowered the risk for MCI and slowed the progression of white matter lesions (WML) [51,52]. Evidence shows that S-equol affects various factors related to ADRD, including arterial stiffness and WML.

### 5.1. Potential Effect of S-equol on Arterial Stiffness

Arterial stiffness is emerging as a high priority as a therapeutic target for AD/ADRD. Arterial stiffness significantly predicts cognitive decline [53,54,55,56,57,58] and incident dementia [59]. Arterial stiffness is also associated with multiple forms of dementia-related pathology, including WML volume, cerebral microbleeds, amyloid-β deposition in the brain, phosphorylated tau, and neuroinflammation in the cerebral spinal fluid [60,61,62,63]. We recently conducted a systematic review and meta-analysis of eight RCTs of soy isoflavones on arterial stiffness [64]. Arterial stiffness was assessed with carotid-femoral pulse wave velocity (PWV), brachial-ankle PWV, and Cardio Ankle Vascular Index (CAVI), all of which are currently widely used in clinical practice and research [58] and with other methods. Supplementation of soy isoflavones significantly improved arterial stiffness compared to placebo (standardized mean difference −0.33, 95% CI: −0.47, −0.19). Because of the short intervention period (24 h to 12 weeks) and the small number of participants in each study (*n* = 17 to 213), trials with longer intervention duration and a larger number of participants are imperative to determine whether supplementation of soy isoflavones improves arterial stiffness.

Three trials directly tested whether supplementation of S-equol improved arterial stiffness. Usui et al. conducted a crossover RCT of 10 mg/day of S-equol supplementation for 12 weeks among 54 middle-aged overweight or obese men and women with metabolic syndrome and on arterial stiffness by CAVI [32]. Supplementation of S-equol significantly improved arterial stiffness (change in CAVI: 0.1 ± 0.1 for placebo and −0.2 ± 0.1 for equol, *p* < 0.01). Yoshikata et al. conducted a single-arm study of 10 mg/day of S-equol supplementation for 12 months among 74 middle-aged women [65]. They assessed arterial stiffness by brachial-ankle PWV. They observed a significant reduction in arterial stiffness (1402 cm/s at baseline and 1376 after 12 months, *p* < 0.01). Yoshitaka et al. more recently conducted an RCT to study the effect of 10 mg/day of S-equol supplementation on arterial stiffness by brachial-ankle PWV for 3 months among 57 postmenopausal women [66]. They did not observe a significant difference in the change in brachial-ankle PWV between the intervention and control groups, likely due to the small sample size. However, after the 3-month intervention, they observed that significantly more women in the control group were found to have worse brachial-ankle PWV than those in the intervention group (26.7% vs. 7.3%, *p* < 0.04). Since arterial stiffness increases monotonically with age [67], these results suggest that supplementation of S-equol inhibits the progression of arterial stiffness. RCTs of S-equol and arterial stiffness with longer duration and a larger number of participants are warranted.

Major underlying mechanisms of arterial stiffness include pathological collagen production and elastin degradation in the arterial wall, endothelial dysfunction, oxidative stress, and inflammation [58]. Preclinical studies show that S-equol inhibits abnormal collagen synthesis and elastase, improves endothelial function, and has antioxidant and anti-inflammatory properties (Table 1). Dubey et al. reported that in human aortic smooth muscle cells, phytoestrogens, including S-equol, dose-dependently inhibited collagen synthesis, proliferation, and migration of smooth muscle cells [68]. This effect is partly mediated through estrogen receptors (ERs) and possibly the direct inhibition of mitogen-activated protein kinase (MAPK). Gopaul et al. reported that treatment with equol decreased the expression of the elastase enzyme by 35% in 8-week organotypic cell cultures of human dermal fibroblasts [69]. This effect is through ERβ. We recently conducted a systematic review on the potential cardioprotective effects of S-equol and showed that S-equol improves endothelial function and possesses antioxidant and anti-inflammatory properties in preclinical studies [70].

### 5.2. Inverse Association of S-equol with White Matter Lesions (WMLs)

WMLs are a significant risk factor for age-related cognitive decline and ADRD [78,79]. The SPRINT-MIND MRI sub-study reported that progression of WMLs was significantly slower in individuals treated with more aggressive blood pressure (BP) control, although the effect size is relatively small, i.e., a mean between-group difference for change in WML volume of −0.54 cm^3^ (95% CI: −0.87 to −0.20 cm3) [51,52]. We recently reported a longitudinal association of S-equol-producing status with WML% (WML volume divided by total brain volume) [80]. This study was nested within a prospective cohort study in Japan [81]. Normal cognition was defined as ≤1 abnormal test result over all domains of the neuropsychological test battery and 91 cognitively normal older adults (45 men and 46 women, mean age of 82) underwent structural brain MRI. Blood levels of S-equol and soy isoflavones were measured using serum samples collected and stored 6–9 years before the imaging study. Soy isoflavones were detected in the blood of all 91 participants and 45 participants (49%) were non-producers. We divided equol producers into a high or low category using the median. Compared to high equol producers, non-producers had >100% higher age- and sex-adjusted WML% (1.19% (95% CI: 0.97, 1.49), 0.89% (95% CI: 0.67, 1.17), and 0.58% (95% CI: 0.44, 0.72) for non-producers, low and high producers, respectively, *p* for trend <0.01). The significant inverse association remained even after additionally adjusting for hypertension, diabetes, dyslipidemia, apolipoprotein E4 (ApoE4), and years of education. Sex-stratified analysis showed that S-equol-producing status was significantly inversely associated with WML% in both sexes. Of note, serum levels of soy isoflavones were not significantly associated with WML%, suggesting that S-equol but not soy isoflavones may be a strong determinant of the progression of WML volume.

Studies consistently associate [62,82] higher arterial stiffness with WMLs in older adults, independent of BP and other cardiovascular risk factors, suggesting that the S-equol effect on arterial stiffness may be one potential mechanism. In addition, a recent meta-analysis in human studies reported a significant inverse association of WMLs with cerebral blood flow [83].

A preclinical study reported that equol increased cerebral blood flow and had vasorelaxant effects on the cerebral artery. Yu et al. investigated the impact of equol on cerebral blood flow and the underlying molecular mechanisms [84]. Single-dose intraperitoneal injection of equol (both S-equol and R-equol) in adult male Sprague-Dawley rats resulted in a significant dose-dependent increase in regional cerebral blood flow in the parietal lobe without significant changes in BP. They also showed that vasorelaxation induced by equol was endothelium independent through activation of large-conductance Ca^2+^ activated K+ channels in the vascular smooth muscle cells. Jackman et al. reported that using adult normotensive and hypertensive Sprague-Dawley rats, equol displayed vasorelaxant activity both in normotensive and hypertensive rats [85]. They also showed that vasorelaxant responses of equol were independent of the endothelium, nitric oxide synthase, or K+ channels. S-equol is reported to improve endothelial function in human endothelial cells of the umbilical vein [74,75,76,77], aorta [77], and pulmonary artery [86]. Still, no study has examined the effect of S-equol on brain microvascular endothelial cells [87].

### 5.3. Other Neuroprotective Properties of S-equol

#### 5.3.1. Antioxidant Properties

Oxidative stress is generally caused by an imbalance between the production and accumulation of reactive oxygen species (ROS) in cells and tissues [88]. Mitochondria mainly produce ROS during both physiological and pathological conditions. ROS accumulation exceeds antioxidant defense and is referred to as oxidative stress [89]. The brain is vulnerable to oxidative stress due to high oxygen consumption, polyunsaturated fat-rich content, and high iron content. Oxidative stress is associated with various pathways leading to neurodegeneration and AD [90,91,92], including amyloid-β accumulation [93,94], mitochondrial dysfunction and apoptosis [93,94,95], cell membrane damage, protein damage [96], DNA damage [96], and neuroinflammation [95].

S-equol has the highest antioxidant properties among all soy isoflavones and their metabolites [20,38]. Antioxidant properties of S-equol are greater than vitamins C and E in in vitro studies [38,97]. Lie et al. showed in deoxycorticosterone acetate-salt-induced hypertensive rats treated with low- (10 mg/kg/BW) and high- (20 mg/kg/BW) S-equol for 4 weeks that S-equol improved both long- and short-term memory and that S-equol increased antioxidant activity in the brain by elevating the activities of superoxide dismutase (SOD), catalase and glutathione peroxidase (GPx) levels and decreasing malondialdehyde (MDA) content and acetylcholinesterase (AChE) activity [98]. Ma et al. showed in male and ovariectomized female Sprague-Dawley rats fed with S-equol (250 ppm), genistein (500 ppm), or isoflavone-reduced diet for two weeks before 90 min transient middle cerebral artery occlusion followed by reperfusion that both S-equol and genistein reduced infarct size and that S-equol and genistein reduced oxidase activity of nicotinamide adenine dinucleotide phosphate and superoxide levels in the rat brain [99]. Furthermore, S-equol reduced plasma thiobarbituric acid reactive substances, a by-product of lipid peroxidation and a biomarker of oxidative stress. Yu et al. examined whether S-equol confers neuroprotection against hypoxia injury by inhibiting the generation of ROS [100]. Using a rat pheochromocytoma cell line exposed to hypoxia injury, they showed that pretreatment with S-equol dose-dependently restored the cell viability and decreased MDA content and lactate dehydrogenase (LDH) activity.

#### 5.3.2. Anti-Inflammatory Properties

Microglia and astrocytes are key regulators of the central nervous system inflammatory responses [101]. Microglial cells are principally responsible for immune defense in the central nervous system. Under pathological conditions, microglial cells are over-activated and produce a variety of proinflammatory cytokines, which can cause neuroinflammation and, subsequently, neurodegeneration. Astrocytes are active players in neuroinflammation and their response may be beneficial or detrimental depending on the stimuli offered by the inflamed milieu [102].

Subedi et al., using microglia and astrocyte cell lines, studied the anti-neuroinflammatory and neuroprotective effects of equol [103]. Equol (both S- and R-equol) inhibited the lipopolysaccharide (LPS)-induced activation of toll-like receptor 4 (TLR4), MAPK, nuclear factor kappa B (NF-κB)-mediated transcription of inflammatory mediators, production of nitric oxide (NO), secretion of tumor necrosis factor-α (TNFα), and interleukin 6 (IL-6). In addition, equol protects neurons from neuroinflammatory injury through the downregulation of neuronal apoptosis. Equol increased the production of nerve growth factors in astrocytes. Moriyama et al., using astrocytes stimulated by LPS, reported that S-equol attenuated LPS-induced increase in NO with a concomitant decrease in expression of inducible NO synthase [104]. This effect was partially mediated via the G protein-coupled receptor 30 on the cell surface. Lu et al. reported in an LPS-induced depression model in mice that S-equol significantly decreased the levels of pro-inflammatory cytokines (TNFα, IL-6, IL-1β) in the hippocampi [105]. In addition, S-equol significantly upregulated the expression of synaptic plasticity-related proteins and downregulated TLR4 and NF-κB signaling pathway in the hippocampi of LPS-treated mice.

#### 5.3.3. Reduction in Amyloid-β Induced Neurotoxicity and Tau Phosphorylation

Major neuropathological hallmarks of AD involve protein aggregation as amyloid-β in extracellular plaques and phosphorylated tau in intracellular neurofibrillary tangles [106]. Several studies reported neuroprotective effects of genistein on amyloid-β-induced neurotoxicity and tau phosphorylation [107,108,109,110,111,112]. Compared to genistein, a few studies have investigated the effect of S-equol on amyloid-β-induced neurotoxicity and tau-phosphorylation. Using SH-SY5Y neuroblastoma cells exposed to amyloid-β, Tsai et al. examined the effect of S-equol and 17β-estradiol [113]. Exposure to amyloid-β reduced cell survival and caused downregulation of steroid receptor coactivator-1 (SRC-1) and extracellular signal-regulated kinase (ERK)1/2. Pretreatment with S-equol or 17β-estradiol reversed these effects of amyloid-β. ER agonists blocked the effects of both S-equol and 17β-estradiol. Using cultured hippocampal neurons, Zhao et al. investigated whether phytoestrogens, including equol and genistein, exert neuroprotective or neurotrophic effects [114]. They showed that following exposure to neurotoxic agents, including amyloid β25-35, phytoestrogens significantly reduced a marker of membrane damage but were ineffective in preventing a decline in neuronal mitochondrial viability. Hirohata et al. investigated the anti-fibrillization, anti-oligomerization, and fibril-destabilizing effects of genistein, glycitein, and equol on Aβ1-40 and Aβ1-42 levels in vitro using nucleation-dependent polymerization monitored by thioflavin T fluorescence, atomic force microscopy, electron microscopy, and photo-induced cross-linking [115]. They showed that glycitein interacted with Aβ monomers, oligomers, and fibrils, and the effect of equol was much weaker than glycitein. Using SH-SY5Y neuroblastoma cells cultured with amyloid-β and 1-methyl-4-phenyl-pyridine, Sorensen et al. investigated the effect of equol and genistein on the neurotoxicant-induced elevation of intracellular calcium [116]. The neurotoxicants caused an increase in Ca^2+^ and both equol and genistein attenuated the increase in Ca^2+^.

Since genistein and S-equol are not only structurally similar but also possess similar affinity to ERβ, effects and mechanisms of genistein on amyloid-β and tau phosphorylation may, to some extent, apply to those of S-equol. Okamura et al., using the human cell lines Daudi, Jurkat, U937, and K562, showed that genistein decreased the generation of amyloid-β by downregulation of the transmembrane protein presenilin 1, which is involved in the amyloid precursor protein (APP) cleavage [107]. Li et al., using female ob/ob and control mice fed with a standard diet or diet with genistein for 4 weeks, showed that genistein reduced amyloid-β deposition and the level of hyper-phosphorylated tau in the brain and that genistein increased the expression levels of the neurotrophic factors, nerve growth factor, and brain-derived neurotrophic factors [112]. Shentu et al., using HEK293-T cells co-infected with a cancerous inhibitor of protein phosphatase 2A (CIP2A) and APP plasmids or CIP2A and tau plasmids, investigated the inhibitory effects of genistein on APP/tau phosphorylation [111]. They showed that genistein significantly reduced APP/tau phosphorylation and amyloid-β production. Pierzynowska et al., using a streptozotocin-induced rat model of the sporadic form of AD, investigated the effect of genistein and showed that genistein degraded amyloid-β and phosphorylated tau protein in the brain [109]. Using retinoic-acid differentiated SH-SY5Y cells treated with amyloid-β25-35, Petry et al. examined the effect of genistein against amyloid-β-induced cell death and underlying mechanisms [108]. Genistein partially inhibited amyloid-β-induced cell death, primarily apoptosis, and the protective effect of genistein was associated with inhibition of amyloid-β-induced Akt inactivation and tau hyperphosphorylation. In another study using male Wistar rats with a bilateral intracerebroventricular infusion of amyloid-β1-42, Petry et al. investigated the effect of genistein on memory impairment and its underlying mechanisms [110]. Genistein improved amyloid-β-induced cognitive impairment by attenuating synaptotoxicity, hyperphosphorylation of tau, and inactivating ERK.

#### 5.3.4. Other Reported Mechanisms

Ariyani et al. studied the effect of equol on cell differentiation and proliferation, using a mouse primary cerebellar culture, Neuro-2A clonal cells, and an astrocyte-enriched culture [117]. S-equol increased the dendrite arborization of Purkinje cells induced by triiodothyronine and the neurite growth of Neuro-2A cell differentiation. This effect was mediated through both ER and GPR30. On the other hand, in astrocytes, S-equol induced cell proliferation and migration, which was mediated through GPR30.

Using a non-contact co-culture model with LPS-BV2 conditioned media and human neuroblastoma SH-SY5Y cells, Johnson et al. demonstrated that S-equol had cytoprotective effects by decreasing LPS-BV2-conditioned media-induced cytotoxicity in SH-SY5Y cells and that S-equol had neuroprotective effects by reducing Parkinson’s disease-related neurotoxin (6-hydroxydopamine and 1-methyl-4-phenylpyridimium)-induced cytotoxicity in SH-SY5Y cells. [118]

## 6. ERβ and Cognition

ERβ is part of the steroid-activated nuclear receptor family of transcription factors and is present in several tissues throughout the body [119,120]. While ERβ density decreases with age, there is no change in estradiol affinity with aging. In contrast, ERα decreases in several receptors and is responsive with age [121]. ERβ is the predominant ER expressed in the brain. It is highly expressed in the hippocampus, cerebellum, and frontal cortex, with an essential role in learning and memory and promoting synaptic plasticity [122,123,124]. While ERα and ERβ mediate the effects of estradiol on normal brain function, ERβ has a more substantial influence on areas related to cognition [122,125].

Single nucleotide polymorphisms (SNPs) in the intragenic region of ESR2, the gene that encodes ERβ, are related to cognitive performance. Some minor alleles are associated with decreased memory and poor executive function, whereas major alleles have cognitive-domain-specific protection [126]. A study in women aged 54–55 found that ESR2 SNPs were associated with decreased semantic memory, as measured by the Boston Naming Test. The addition of the ApoE4 allele to the analyses demonstrated that the effects of ESR2 SNPs on executive function were dependent on the ApoE4 allele, where ESR2 SNP-related impairments in semantic memory were ApoE4 allele independent [126]. Further, ESR2 polymorphisms influence the incidence of AD [127]. Thus, through variations in ESR2 SNPs, ERβ plays a crucial role in neurocognitive health and increasing AD susceptibility.

ERβ activation improves performance in hippocampus-dependent memory tasks and increases recognition memory through activation of the pre-frontal cortex and hippocampal signaling pathways [123,128]. ERβ is implicated in transcriptional regulation of genes involved in the maintenance of hippocampal function with aging and expression of phosphorylated MAPK/ERK, which is implicated in AD development [129,130]. ERβ stimulation induces protein kinase B (Akt) and tropomyosin receptor kinase B (TrKB) expression, which are involved in hippocampal-dependent behaviors, such as memory and cognition [131]. Further, studies with ERβ knockout mice found impaired spatial learning and severely disrupted memory behaviors [132]. ERβ was found to play a role in cAMP response element-binding protein (CREB) expression and phosphorylation, a cellular transcription factor involved in learning and long-term memory [128,133]. In the cerebellum, ERβ plays a crucial role in motor learning by potentiating neuronal plasticity and synaptogenesis [134]. As ERβ promotes estrogen-mediated neuronal plasticity and memory function, selective targeting of ERβ may help maintain cognitive function and decrease the incidence of AD.

## 7. Neuroprotection by ERβ Agonists

ERs are intracellular transcription factors. Both ERα and ERβ are expressed in the brain and cardiovascular system [8]. ERα is predominantly present in the mammary gland, uterus, bone, and adipose tissue, while ERβ is mainly expressed in lung, prostate, immune cells, and adipose tissues.

ERβ has a minor role in the proliferation of the mammary gland or endometrium. Thus, selectively targeting ERβ is an attractive way to harness the beneficial effects of ER while reducing unwanted adverse effects. For this reason, pharmaceutical companies and academic institutes have developed ERβ agonists as potential treatment agents for cancer, cardiovascular disease (CVD), and neurological disorders, including ADRDs [135]. Table 2 summarizes ERβ agonists that have been developed for neuroprotection.

Diarylproprionitrile (DPN) was the first ERβ agonist to be discovered. DPN has a 70-fold ERβ selectivity over ERα in the binding assay. In preclinical studies, DPN improved maintenance of cognitive function, suppressed anxiety-like behaviors, and reduced neuroinflammation, pro-inflammatory cytokines, and neural cell death [136,137,138,139,140,141].

PhytoSERM is a combination of genistein, daidzein, and equol and has an 83-fold ERβ selectivity over ERα [145]. Preclinical studies show that PhytoSERM potentiated mitochondrial bioenergetics and increased the expression of mitochondrial antiapoptotic proteins and amyloid-β degrading enzymes [144,145]. In addition, in a female triple-transgenic mouse model of AD, 9-month treatment with PhytoSERM promoted physical health, improved spatial recognition memory, and attenuated amyloid-β deposition in the brain compared to controls [142]. Among ERβ agonists listed in Table 2, PhytoSERM is the only one tested in humans. An RCT of 50 mg/day, 100 mg/day, or placebo for 12 weeks among 71 women aged 45–60 years was conducted to assess the safety and tolerability of PhytoSERM [142]. A retrospective responder analysis of the trial showed that 50 mg/day of PhytoSERM significantly preserved cognitive function in areas of verbal learning and executive function [143]. An RCT of PhytoSERM among pre- and peri-menopausal women will soon start (R01 AG075122). This RCT aims to determine the efficacy of PhytoSERM to sustain glucose metabolism (because the menopausal transition is accompanied by reduction in cerebral metabolic rate of glucose, which correlates with progression of AD biomarkers later in life), on cognitive function and other outcomes.

Silibinin is an active milk thistle extract and is a selective ERβ agonist. Silibinin is used to treat toxic liver damage and is an adjunctive therapy for chronic hepatitis and cirrhosis [154]. Preclinical studies show that silibinin protects against amyloid-β-induced anxiety/depression-like behavior [147] and memory deficit [148]. EGX358 or ISP358-2 has a 750-fold selectivity for ERβ over ERα in a cell-based assay. Preclinical studies showed that EGX358 improved memory [150,151]. 8β-VE2 has a 183-fold ERβ selectivity in the transactivation assay [155]. Injection of 8β-VE2 in aromatase knockout mice prevented dopaminergic cell death in the medial preoptic area [152]. SERM-beta1 and SERM-beta2 are derivatives of tetrahydorfluorenose and have a 129-fold ERβ-selective ligand. Treatment with these agents for 4 days in ovariectomized rats showed an increase in the total number of cells in the dentate gyrus of the hippocampus [153].

## 8. Conclusions

It has long been hypothesized that S-equol producers, compared to non-producers, have more health benefits from consuming soy isoflavones [20], including alleviating postmenopausal symptoms, bone health, breast and prostate cancers, CVD, and cognitive decline. Some but not all observational studies have supported this hypothesis [30,35]. Inconsistent results in these observational studies may partly be due to changes in S-equol-producing status over time [156,157]. In contrast, although evidence from preclinical studies supports health benefits of S-equol, as reviewed above, the possibility cannot be ruled out that some phenotypes associated with S-equol producers bring these health benefits.

S-equol is available as a dietary supplement and seven RCTs of S-equol have been conducted and reported [32,66,158,159,160,161,162]. Almost all these trials investigated the effect of S-equol on menopausal-related symptoms with short durations of intervention (8 to 12 weeks). An RCT of S-equol supplementation for 24 months among 400 older adults without dementia will soon start (R01 AG074971). This trial will answer whether a 24-month supplementation of S-equol will significantly improve arterial stiffness, slow the progression of WML volume and cognitive decline, and whether S-equol itself or phenotypes associated with equol producers result in such benefits.

## Figures and Tables

**Table 1:** Potential mechanisms of S-equol to slow the progression of arterial stiffness (image of the white matter lesion is from Wikimedia Commons; images of amyloid β plaques and a cross-sectional view of the aorta were created with BioRender).


Arterial stiffness is an emerging target for preventing cognitive decline and dementia
• Emerging as a potential therapeutic target for preventing vascular contribution of cognitive impairment and dementia (VCID) and Alzheimer’s disease and related dementias (ADRD) [58,71]. • Significantly associated with cognitive decline [53,54,55,56,57,58]. • Signifincatly associated with multiple forms of dementia-related pathology, including white matter lesion volume and Amyloid-β deposition in the brain [53,54,55,56,57,58]. • Significantly associated with incident dementia in some [59] but not all [72,73] prospective cohort studies. 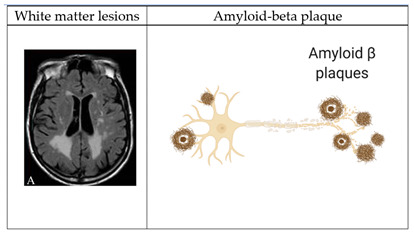
Pathophysiology of arterial stiffness
Major underlying mechanisms of arterial stiffness include pathological collagen production and elastin degradation in the arterial wall, endothelial dysfunction, oxidative stress, and inflammation [58]. 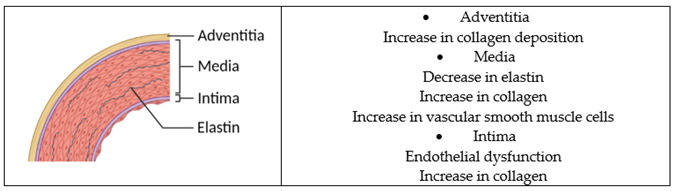
Potential mechanisms of S-equol that slow the progression of arterial stiffness
• S-equol inhibits abnormal collagen synthesis, proliferation, and migration of smooth muscle cells in the human aorta [68]. • S-equol may inhibit the degradation of elastin in the arterial wall by decreasing the expression of the elastase enzyme [69]. • S-equol improves endothelial function in the human aorta and umbilical vein endothelial cells [74,75,76,77]. • S-equol possesses anti-inflammatory and antioxidant properties [70].

**Table 2:** Estrogen receptor β agonists are being investigated for neuroprotection.

Name	Findings
Diarylproprionitrile (DPN)	Treatment with DPN-protected rat cultured hippocampal neurons from glucose deprivation-induced cell death [136]. Treatment with DPN of chronic experimental autoimmune encephalomyelitis mice (transgenic proteolipid protein-enhanced green fluorescent protein transgenic mice in the C57BL/6J background) prevented histopathological (fewer demyelinated, damaged axons and more myelinated axons) and increased mature oligodendrocyte numbers [137]. Pretreatment with DPN decreased neuronal cell death by reducing reactive oxygen species in Aβ1-42 induced oxidative stress and inflammation in primary rat cortical cell culture [138]. In addition, DPN pretreatment decreased pro-inflammatory cytokines (IL-1β and IL-6) In C57/B1/6 female mice performed with spinal cord injury, the administration of DPN reduced neural apoptosis and inflammation compared to untreated groups [139].In middle-aged female Harlan-Wistar rats with ovariectomy followed by no replacement or replacement with estradiol, DPN, or LE2 (ERα agonist), gene expression related to the innate immune system, specifically macrophage-associated and complement genes, in the hippocampus was investigated. Ovariectomy caused the upregulation of these gene expressions, and estradiol, DPN, and LE2 attenuated the increase in these gene expressions [140]. In middle-aged female Harlan-Wistar rats implanted subcutaneously in the neck with DPN or vehicle only for 29 days, the effect of DPN on hippocampal transcriptome was investigated. DPN contributed to regulating transcription, translation, neurogenesis, neuromodulation, and neuroprotection in the hippocampal formation [141].
PhytoSERM	A first human study of PhytoSERM-Phase 1b/2a randomized, double-blind placebo-controlled study of 50 or 100 mg/day PhytoSERM for 12 weeks, recruiting 71 peri-menopausal women aged 45–60, showed that both 50 and 100 mg/day were well tolerated but based on safety outcomes, 50 mg/day was considered preferable [142]. A retrospective responder analysis of the trial mentioned above showed that 50 mg/day of PhytoSERM significantly reduced hot flashes and preserved cognitive function in verbal learning and executive function [143]. In mitochondrial markers in rat hippocampal neuronal cultures and a female mouse ovariectomy model, both S-equol phytoSERM and R/S-equol phytoSERM treatments potentiated mitochondrial bioenergetics [144]. In rat primary hippocampal neuronal cultures challenged with neurotoxic glutamate or amyloid-β_1–42_, phytoSERM showed a greater effect on neuronal survival than genistein, daidzein, equol, or IBSO03569. In ovariectomized adult female rats, treatment with phytoSERV significantly enhanced brain mitochondrial bioenergetics. Furthermore, in western blot analyses of hippocampal protein, phytoSERM increased expression of brain mitochondrial anti-apoptotic protein Bcl-2 and Bcl-xL as well as insulin-degrading enzyme and neprilysin, which are amyloid-β degrading enzymes [145]. A 9-month supplementation of phytoSERM in a female triple transgenic mouse model of Alzheimer’s disease promoted physical health, prolonged survival, improved spatial recognition memory, and attenuated amyloid-β deposition and plaque formation in the brain [146].
Silibinin	Oral gavage of silibinin protected amyloid-β_1–42_ induced anxiety/depression-like behaviors in male Sprague-Dawley rats. Silibinin significantly suppressed neuronal damage induced by amyloid-β_1–42_ in hippocampus regions, increased brain-derived neurotrophic factor, and attenuated the increase of autophagy levels in the hippocampus [147]. Silibinin-protected amyloid-β_25–35_ induced memory deficits in male Sprague-Dawley rats. Silibinin increased the expression of anti-inflammatory cytokine (interleukin 4) and decreased the expression of pro-inflammatory cytokines (interleukin 1β) in the hippocampus. Silibinin also increased glutathione levels and decreased malondialdehyde levels, indicating antioxidant properties of silibinin [148]. Treatment with silibinin of astrocyte induced with lipopolysaccharide decreased the reactive form of astrocyte, attenuated nitrite release, increased glutathione levels, reduced malonaldehyde, and reduced glial fibrillary activated protein [149].
EGX358 or ISP358-2	Intrahippocampal infusion or intraperitoneal injection of ISP358-2 enhanced memory consolidation in C57BL/6 ovariectomized female mice [150]. EGX358 reduced drug-induced vasodilation and enhanced spatial and object recognition memory without adversely affecting anxiety- or depression-like behaviors in C57BL/6 ovariectomized female mice gavaged with EGX358 for 9 weeks [151].
8β-VE2	Injecting 8β-VE2 for 6 weeks in aromatase knockout male mice prevented dopaminergic cell death in the medial preoptic area but not in the rostral and medial arcuate nucleus [152].
SERM-beta1 and SERM-beta2	Treatment with SERM-beta 1 or SERM-beta 2 for 4 days in ovariectomized Sprague-Dawley rats stimulated an increase in the total number of cells in the dentate gyrus of the hippocampus [153].

## Data Availability

Not applicable.
